# The Depression Schema: How Labels, Features, and Causal Explanations Affect Lay Conceptions of Depression

**DOI:** 10.3389/fpsyg.2015.01728

**Published:** 2015-11-17

**Authors:** Paul H. Thibodeau, Mira J. Fein, Elizabeth S. Goodbody, Stephen J. Flusberg

**Affiliations:** ^1^Department of Psychology, Oberlin College, OberlinOH, USA; ^2^Department of Psychology, State University of New York at PurchasePurchase, NY, USA

**Keywords:** schemas, depression, framing, folk psychiatry, concepts

## Abstract

Depression is a common clinical disorder characterized by a complex web of psychological, behavioral, and neurological causes and symptoms. Here we investigate everyday beliefs and attitudes about depression, as well as the factors that shape the depression schemas people hold. In each of three studies, participants read about a person experiencing several symptoms of depression and answered questions about their conception of the disorder. In some cases the symptoms were presented in isolation while in other cases the symptoms were presented with a diagnostic label and/or descriptions of its possible causes (e.g., genes versus personal experience). Results indicated that beliefs and attitudes toward depression were largely shaped by individual difference factors (e.g., personal experience, political ideology) and that the experimental manipulations primarily impacted attributions of responsibility and suggestions for a course of treatment. These findings represent an important advance in our understanding of the factors that influence the folk psychiatry of depression and help inform theories of schema formation for abstract and complex domains.

## Introduction

People use schematic knowledge structures to organize, interpret, and represent information about the world around them (Bartlett, 1932; Minsky, 1975; Rumelhart, 1975; Schank and Abelson, 1975; Bransford, 1979). In one classic demonstration, people were better able to understand and remember a description of a procedure for doing the laundry when they were told ahead of time it was about “washing clothes" (i.e., when they had activated the relevant schema) than when they were not given any contextual cues (Bransford and Johnson, 1972). This experiment, and many others, illustrate that conceptual knowledge is richly structured and not simply an amalgam of disconnected facts.

Decades of research have addressed questions of how schematic knowledge is learned, retrieved, and used (e.g., Mandler, 1984; Rogers and McClelland, 2004). This work has identified nuanced factors that moderate how information is weighted and deployed (e.g., certain domains prioritize superficial features, whereas other domains are organized around unobservable “essences”; e.g., Gelman and Wellman, 1991). For instance, in some cases manipulating a single word or short phrase (i.e., linguistic framing) can change the schema that people bring to mind and, in turn, change how people respond to information they are given (e.g., Kraut, 1973; Loftus, 1975; Tversky and Kahneman, 1981; Walton and Banaji, 2004; Bryan et al., 2011; Thibodeau and Boroditsky, 2011, 2013, 2015); in other cases, more elaborate manipulations are required to instantiate and transfer a relevant schema for use in solving a target problem (see, e.g., Gick and Holyoak, 1980, 1983).

In the present paper, we investigate several fundamental issues about the nature of schematic knowledge for depression. Although the studies are designed around this singular domain, at the heart of our investigation are theoretical questions about the nature of schematic knowledge. For example, to what extent do different labels (“depression” versus “neurological disorder”) and causal explanations (experience versus genes) for the condition affect the way people conceptualize a person’s struggle with depression (e.g., stigmatizing attitudes, beliefs about the biological basis of the condition, etc.)?

One reason we focus our investigation on depression is that psychological disorders (as well as other mental states, emotions, and psychological constructs) are difficult to classify along dimensions that have been studied more extensively in the cognitive sciences. On one hand, these domains are fairly abstract in that they are not merely experienced through the senses (e.g., touched, seen, smelled) and therefore do not fit within taxonomies of concrete things (e.g., artifacts versus natural kinds; Gelman and Wellman, 1991). On the other hand, these domains are also somewhat different from classic examples of abstract concepts (e.g., time, love, justice), at least for people who have not suffered from depression, for which media and language (e.g., metaphor) may be the primary source of information about the subject (e.g., Lakoff and Johnson, 1980; Boroditsky, 2000). Indeed, the variability with which people have personal experience with conditions like depression represents an important opportunity for research on schematic knowledge in cognitive science: to what extent does the subjective experience of depression (e.g., symptoms, treatments, and discussions of the disorder with friends, family, and health professionals) influence conceptions of the disorder?

The present work seeks to understand the role of language and causal explanations for depression. It builds on work on conceptual knowledge in cognitive science as well as debates on labeling in the context of psychiatric disorders (e.g., Goffman, 1963; Scheff, 1966; Gove, 1975; Ahn et al., 2009; Dar-Nimrod and Heine, 2011; Link and Phelan, 2013). In the context of mental health, some have argued that labels carry a rich structure of associations that can promote stereotyping and stigma (e.g., Scheff, 1966). Evidence for such a view can be seen in anecdotes from patients who describe their experience dealing with a diagnosis (e.g., “it is important to understand that we are faced with recovering not just from mental illness, but also from the effects of being labeled mentally ill,” Deegan, 1993, p. 10). However, others have argued that labels have minor effects on how people think about mental illness and instead suggest that symptoms and symptomatic behaviors cause people to develop stigmatized attitudes toward disorders (e.g., Gove, 1975).

Given this debate, our focus on depression is also motivated by practical concerns. Depression is among the most prevalent mental disorders, affecting more than 15 million Americans every year, and is a leading cause of disability claims worldwide (World Health Organization [WHO], 2012). In recent years, arguments over how to label mental disorders have resurfaced, motivated by a desire to reduce stigmas associated with mental health issues that represent a primary barrier to treatment (Us Department of Health and Human Services, 1999). Many have pointed to labels that emphasize the neurobiological component of mental illness as tools for promoting a view that mental disorders are similar to other less stigmatized “brain diseases” (Sartorius, 1997; US Department of Health and Human Services, 2003). On this view, describing depression in terms of neurobiological processes is thought to highlight the fact that mental disorders have physiological markers and are caused by factors outside a person’s control.

Data from the General Social Survey suggests that messaging campaigns that emphasize the neurobiology of depression have increased recognition of the condition’s physiological underpinning (Pescosolido et al., 2010). For instance, between 1996 and 2006 there was a significant increase in the percentage of the population who viewed depression as resulting from neurobiological causes (67% compared to 54%), as well as increased support for depression-related treatment (Blumner and Marcus, 2009). However, somewhat paradoxically, emphasizing the neurobiological component of mental illness does not seem to have reduced the stigma associated with depression (Pescosolido et al., 2010).

In fact, some recent research suggests that biological accounts of psychopathology can actually increase stigma – by making patients seem even more different from the rest of the population and by increasing prognostic pessimism (Phelan et al., 2006; Deacon and Baird, 2009; Dar-Nimrod and Heine, 2011; Lebowitz et al., 2013; Lebowitz and Ahn, 2014). Therefore, it is important to rigorously assess how factors such as labeling influence how people think and reason about depression and other psychological disorders.

### The Present Study

In three experiments we measure (a) people’s predisposition to use psychological or neurobiological labels to characterize depressive symptoms, (b) the associations that people have with psychological and neurobiological labels for extended descriptions of depressive symptoms, and (c) the causal power of such labels and explanations to affect attitudes and conceptions of depression.

We considered two hypotheses. One possibility, in line with the recommendations put forward by public health officials, is that emphasizing the neurobiological component of depression (e.g., calling it a “neurological disorder”) will lead people to adopt a more compassionate view of those suffering from depression (e.g., US Department of Health and Human Services, 2003). Recognition of the neurobiological underpinning of depression may decrease stigma toward the disorder, lead people to feel more empathy with people suffering from the condition, and promote the view that depression is a disease. In contrast, a second possibility is that emphasizing the neurobiological component of depression will not lead people to change their view of the disorder or that it will actually increase people’s tendency to stigmatize those suffering from depressive symptoms. For instance, prior work has found that clinicians conceptualize mental health disorders along a single continuum from the highly biological to the highly psychological (non-biological or behavioral; Ahn et al., 2009) and that using biological labels to describe a given mental disorder (e.g., schizophrenia, depression, obsessive–compulsive disorder) leads mental health clinicians to empathize less strongly with patients suffering from the conditions (Lebowitz and Ahn, 2014).

These accounts are contrasted in three experiments. In Experiment 1 we presented participants with a narrative that described a person suffering from depressive symptoms and prompted them for a “diagnosis” for the protagonist (as *normal*, a *neurological disorder*, or *depression*). We also gaged various attitudes related to depression. In Experiment 2, we manipulated the label used to describe a person experiencing depressive symptoms – framing the protagonist’s experience as resulting from “depression” or a “neurological disorder” (we also included a condition in which the symptoms were described without a label). In Experiment 3, we included a more elaborate description of the physiology of depression and identified either genes or a negative life experience as a causal contributor to a person’s experience of depression. This allowed us to test whether extended descriptions and causal explanations would be more effective than subtle labeling manipulations in changing someone’s beliefs about depression.

Of note, we also manipulated whether the protagonist in the narrative was male or female. Depression disproportionately affects women (Kuehner, 2003), and therefore people’s expectations (prior beliefs) about whether a pattern of symptoms might be indicative of clinical depression may depend on whether the individual is male or female. People also hold different stereotypical assumptions about the emotional lives of men and women, and so might judge the same set of behaviors differently depending on whether they conform to or violate gender norms.

We expected that regardless of the label or explanation’s effect on stigma and empathy, emphasizing the neurobiological component of depression would affect judgments of how the condition should be treated – with medication rather than psychotherapy (cf. Ahn et al., 2009). We were also interested in whether variability in patterns of diagnosis (Experiment 1), the manipulation of the label (Experiment 2), or the inclusion of a causal explanation (Experiment 3) would affect the degree to which participants empathized with the protagonist, attributed responsibility to him or her for their experience of depressive symptoms, viewed depression as a disease, and exhibited stigmatized attitudes toward the disorder.

Of note, we also collected several individual difference measures and predicted that these factors (personal history with depression, gender, political ideology) would influence how people responded to the description of the protagonist. Specifically, we predicted that liberals, people with more education, women, and those with a personal experience with depression would be more likely to view the condition as a disease, more likely to empathize with those suffering from depression, and more likely to suggest medical treatment options (Skitka et al., 2002; Corrigan and Watson, 2007; Lebowitz et al., 2013; Flusberg et al., 2015; Thibodeau et al., 2015).

## Materials and Methods

### Participants

We recruited and paid 200, 900, and 1200 participants in Experiments 1, 2, and 3, respectively, through Amazon’s Mechanical Turk (Buhrmester et al., 2011; Berinsky et al., 2012). All three studies were conducted in accordance with the recommendations of Oberlin College’s Institutional Review Board and the Declaration of Helsinki. All participants indicated their informed consent (by checking a box on the web-based survey) before beginning the study.

We used Mechanical Turk’s exclusion capabilities to ensure that participants lived in the US and had a good performance record on previous tasks. At the end of the survey, participants were assigned a random number to submit into the Turk interface (see **Table 1** for demographic information from all three samples). Data were excluded from analysis if participants did not provide an accurate completion code or if they contributed data to an earlier experiment (e.g., data was omitted from analysis of Experiment 2 from a small number of participants who had participated in Experiment 1).

**Table 1:** Sample information.

	Experiment 1	Experiment 2	Experiment 3
N sampled	200	900	1200
N analyzed	189	847	1102
Gender: male	41%	52%	51%
Age (range: 18–76)	*M* = 36.15 (*SD* = 12.42)	*M* = 33.13 (*SD* = 10.74)	*M* = 31.75 (*SD* = 10.34)
Education level: a least some college	88%	90%	88%
Democrats	44%	47%	42%
Independents	31%	29%	41%
Republicans	25%	11%	17%
Ideology (100 = very conservative)	*M* = 41.3 (*SD* = 27.5)	*M* = 37.8 (*SD* = 26.4)	*M* = 38.8 (*SD* = 25.7)
Personal history of mental illness	48%	49%	55%
Diagnosed with depression	26%	31%	33%


*Demographic information about the samples for Experiments 1, 2, and 3.*

Determinations of sample size were guided by prior work on similar issues (e.g., Thibodeau et al., 2015) and through the use of a sample size calculator, which was specifically designed to estimate the amount of data needed to construct a structural equation model (Soper, 2015), which is presented in supplementary material. Effect sizes from prior work (e.g., Cohen’s *d* = 0.23 for a difference in empathy associated with the manipulation of a label for depression in Lebowitz and Ahn, 2014) suggest that the studies reported here have statistical power greater than 0.8 to detect possible differences in Experiments 2 (e.g., between the “depression” and “neurological disorder” conditions) and Experiment 3 (e.g., between conditions that included or excluded information about the neurobiological basis of depression).

Of note, all samples included more Democrats and fewer Independents than in the general American population. Recent polling suggests that 32, 39, and 24% of Americans identify as Democrats, Independents, and Republicans, respectively (Pew, 2015).

The proportion of participants who reported a history of mental illness in general and depression specifically is in line with recent national survey data, which estimated a 51% lifetime risk of experiencing at least one anxiety (32%), mood (28%), impulse-control (25%), or substance-abuse disorder (16%; Kessler et al., 2005). The American Psychiatric Association classifies depression primarily as a mood disorder but also considers it to be a component of anxiety, impulse-control, and substance-abuse disorders (American Psychiatric Association [APA], 2013).

### Narrative

In all three experiments, participants first read a description of someone experiencing five symptoms of depression (thereby meeting the diagnostic criteria for clinical depression), taken from the DSM-V (American Psychiatric Association [APA], 2013): trouble sleeping, tiredness, difficulty concentrating, anhedonia, and weight loss. For half of the participants, the protagonist’s name was implied to be female (Jenny); for the other half of participants, the protagonist’s name was implied to be male (Mark).

In Experiment 1, the narrative read:

{Jenny/Mark} is a college student who has recently been having trouble sleeping. S/He has been tired throughout the day, regardless of how long s/he sleeps, and has been having trouble concentrating in class. Activities s/he used to enjoy now feel like a burden on his time. S/He lost a lot of weight recently and doesn’t know why.

In Experiment 2, there were three versions of the narrative (6 when accounting for the gender manipulation): one, the “no label” version, was identical to that of Experiment 1; the other two narratives introduced the protagonist with a label for their symptoms – either “depression” or “neurological disorder” – in the first sentence (e.g., “Jenny/Mark is a college student who has depression. S/He has recently…”).

In Experiment 3, there were six versions of the narrative (12 when accounting for the gender manipulation). Each narrative included the paragraph above (with the “depression” label at the beginning of the report). Half of the participants read additional information about the neurological underpinning of depression adapted from an article on depression (Miller, 2009):

Researchers have found a number of abnormalities in the neurobiology of people with depression. There are at least three brain regions that seem to work differently for people with depression. For instance, the hippocampus tends to be smaller in some depressed people. In addition, receptors of brain cells seem to be oversensitive or insensitive to neurotransmitters, which allow cells to communicate to one another.

Two thirds of participants read about one of two potential causal factors for the protagonist’s depression. One causal factor highlighted the role of genetic heritability in depression:

Several of Mark/Jenny’s relatives also suffer from depression. Researchers have found that depression is 40–50% heritable, suggesting that the cause of depression for many people can be traced to their genes.

A second causal factor highlight the role of personal experience:

Mark/Jenny’s father died when she was young. Researchers have found that profound early losses, such as the death of a parent, may resonate throughout life, eventually expressing themselves as depression.

These variations of the narrative in Experiment 3 can be summarized as a two gender (Mark or Jenny) by two neurological information (present or absent) by three causal information (genetic, experiential, none) between-subjects manipulation.

### Manipulation Check

After reading the narrative, participants were asked to estimate the age, race, gender, and political ideology of the protagonist. Since the report did not suggest answers to questions about the protagonist’s actual age, race or political ideology, these questions were inherently speculative. They were included to partially mask the more relevant question about the protagonists’ gender, which was included as a manipulation check of the gender manipulation. In Experiments 1, 2, and 3, 100, 99, and 97% of participants who read about Mark reported thinking that the protagonist was male, and 100, 96, and 98% of participants who read about Jenny reported thinking that the protagonist was female, respectively.

### Dependent Measures

After participants read the narrative, they were asked to make several judgments about the protagonist, their symptomology, and depression generally. Summary statistics and indicators of reliability for the dependent measures are shown in **Table 2**.

**Table 2:** Dependent measures.

	Experiment 1	Experiment 2	Experiment 3
**Diagnosis (multiple choice)**			
Depression	74%	NA	NA
Neurological Disorder	10%		
Normal	16%		
Seek Help? (% yes)	86%	97%	97%
**Treatment (multiple choice)**			
(a) Counseling	70%	54%	66%
(b) Prescription drugs	4%	4%	2%
(c) Counseling and drugs	17%	28%	24%
(d) Alternative therapies	12%	13%	7%
Empathy (12 items)	*M* = 3.87 (*SD* = 0.53) *α* = 0.768	*M* = 3.87 (*SD* = 0.58) *α* = 0.827	*M* = 3.95 (*SD* = 0.62) *α* = 0.834
Responsibility (3-point scale)	*M* = 1.70 (*SD* = 0.61)	*M* = 1.77 (*SD* = 0.59)	*M* = 1.66 (*SD* = 0.62)
Depression as disease (101-point scale)	*M* = 63.39 (26.92)	*M* = 63.3 (*SD* = 27.74)	*M* = 62.12 (*SD* = 27.57)
Depression Stigma (15 items)	*M* = 1.92 (*SD* = 0.50) *α* = 0.765	*M* = 1.86 (*SD* = 0.55) *α* = 0.820	*M* = 1.92 (*SD* = 0.54) *α* = 0.803


*Summary of dependent measures used in experiments.*

#### Diagnosis

In Experiment 1 (but not Experiments 2 or 3), participants were asked to categorize the protagonist’s symptoms as resulting from “depression,” “a neurological disorder,” or “the normal ups and downs of life” in a multiple choice question.

#### Treatment

In all three experiments, participants were asked whether the protagonist should seek help (“yes” or “no”) and, if so, what kind. There were four treatment options provided that participants could choose between: “counseling,” “prescription drugs,” “counseling and prescription drugs,” or “alternative therapies.” We contrast the two treatment options that included medication (i.e., “prescription drugs” and “counseling and prescription drugs”) with the two treatment options that did not (i.e., “counseling” and “alternative therapies”).

#### Perceptions of the Protagonist

In all three experiments, participants then indicated the extent to which they identified with the protagonist by completing a 10-item empathy scale (e.g., “I can really identify with what was described in the message”; adapted from Campbell and Babrow, 2004). Ratings were made on a 5-point scale that ranged from “Strongly disagree” to “Strongly agree.”

Following prior work on related issues (e.g., on the role of personal narratives in conceptions of obesity; Niederdeppe et al., 2014), we supplemented Campbell and Babrow (2004)’s empathy scale with two additional questions: (1) “How similar do you think you are to this character?” (rated on a 5-point scale from “Very dissimilar” to “Very similar”); and (2) “To what extent do you feel angry or frustrated toward the story’s protagonist?” (rated on a 4-point scale from “Not at all” to “A strong amount of frustration/anger”). For parsimony and clarity, we chose to combine responses to these supplemental questions with responses from the empathy scale (ratings of frustration were reverse scored). The supplemental questions were conceptually related to questions on the empathy scale, reflecting the degree to which participants identified with the protagonist. Combing the empathy scale with the supplemental questions also improved the reliability of the metric: data from each of the three experiments revealed greater internal consistency when the two supplemental questions were averaged with the 10 items from the empathy scale (Chronbach’s *α* increased from 0.734 to 0.768, from 0.823 to 0.843, and from 0.802 to 0.822 in Experiments 1, 2, and 3, respectively).

Participants also indicated how responsible they thought the protagonist was for feeling the way they did by answering the question “How responsible do you think Jenny/Mark is for feeling the way that s/he does?” on a 3-point scale that ranged from “Not at all,” to “Somewhat,” to “Very” responsible. We used a 3-point scale because we anticipated that participants would find it difficult to distinguish between a more subtle range of response options and because prior work has found that 3-point scales can be just as valid and reliable as 5- or 7-point scales (Jacoby and Matell, 1971).

#### Depression

Finally, we measured the extent to which participants considered depression a disease and their feelings of stigma toward the disorder. After recommending a course of treatment and responding to questions about their perception of the protagonist, participants were asked “To what extent do you believe that mental health issues like depression are a lifestyle problem (resulting from a lack of personal self control) versus a disease?” on a 101-point scale where 0 was labeled “personal” and 100 was labeled “disease.” Stigma was gaged with a 15-item inventory (Arbanas, 2008), in which participants rated their agreement with statements like “A person with depression is dangerous and unpredictable” on a 5-point scale that ranged from “Definitely no” to “Definitely yes.” Responses to the stigma scale were aggregated; higher values reflect a more stigmatized view of depression.

#### Individual Difference Measures

After the study, participants were asked a set of background questions, including their personal history of mental illness, age, gender, education level, annual salary, and political ideology (see **Table 1**).

### Data Analysis

To analyze dichotomous responses in all three experiments (i.e., for two dependent measures: whether or not the participant suggested that the protagonist seek help and what type of help the participant suggested that the protagonist seek), we compared the fit of nested logistic regression models. In Experiments 1 and 2 we compared four models: Model 1 did not include predictor variables aside from the intercept; Model 2 included dummy coded predictor variables for the diagnosis (Experiment 1: depression was treated as the “baseline” since it was the modal response) or label (Experiment 2); Model 3 tested for a main effect of the gender of the protagonist and was compared to Model 2 (i.e., controlling for an influence of the diagnosis or label); and Model 4 tested for an interaction between these factors. A similar procedure was followed in Experiment 3, by testing for effects of including neurobiological information about depression, a causal explanation for depression, and the gender of the protagonist. In every analysis, the deviance between the models (i.e., difference in likelihood ratios) is reported as an index of model fit: model deviance approximates a chi-square distribution with the number of added parameters as its degrees of freedom (Menard, 2002).

We report the results of chi-square tests for analyses that involve categorical dependent measures with more than two levels (i.e., the diagnosis question in Experiment 1) and ANOVAs for analyses that involve interval scaled dependent measures.

## Results

### Diagnosing the Symptoms

In Experiment 1, most people (74%) indicated that they thought the protagonist was experiencing depression. Thus people tended to recognize the description of the protagonist as the typical experience of depression. However, there were also a fair number of participants who identified the protagonist’s struggle as part of the normal ups and downs of life (16%) and a small group of participants who conceptualized the symptoms as the result of a neurological disorder (10%). There was no difference in the patterns of diagnosis by the gender of the protagonist, χ*^2^*(2) = 1.66, *p* = 0.436.

Variability in the diagnosis allowed us to explore whether different conceptions of the symptoms were associated with different attitudes toward the protagonist and his or her experience. However, given the relatively small number of participants who diagnosed the protagonist with a neurological disorder (*n* = 17) or as suffering from the normal ups and downs of everyday life (*n* = 30), these analyses are limited in several cases.

### Treatment

In Experiment 1, 86% of participants suggested that the protagonist seek help. People were particularly likely to suggest that the protagonist seek help if they diagnosed him or her with “depression” (98%) or a “neurological disorder” (90%). Fewer participants suggested that the protagonist seek help when the symptoms were thought to be a normal part of life (30%). Logistic regression was used to model treatment suggestions. The analyses revealed that participants suggested that the protagonist should seek help at significantly different rates depending on the diagnosis, χ*^2^*(2) = 72.965, *p* < 0.001 and that there was no effect of the gender of the protagonist on the patterns of diagnosis, *p*s > 0.10. Participants were more likely to suggest a course of treatment that included medication if they thought the protagonist was experiencing a neurological disorder (47%) than if they thought the protagonist was experiencing depression (18%) or the normal ups and downs of everyday life (11%), χ*^2^*(2) = 6.964, *p* = 0.031, which is consistent with prior work (see, e.g., Ahn et al., 2009). There was no effect of the gender of the protagonist on treatment suggestions, *p*s > 0.4.

Given these results, we expected participants in Experiments 2 who were presented with a diagnostic label (“depression” or “neurological disorder”) to be more likely to suggest that the protagonist seek treatment. We also expected differences in the kinds of treatment suggested as a function of the label: a protagonist identified as suffering from a “neurological disorder” may be especially likely to elicit suggestions to seek pharmacological treatment. This is exactly what we found.

In Experiment 2, 97% of participants suggested that the protagonist seek help. A logistic regression revealed that participants were more likely to suggest that the protagonist seek help when the narrative included a label (“depression”: 98%; “neurological disorder”: 99%) than when it did not (94%), χ^2^(2) = 10.130, *p* = 0.006; there was no effect of the gender of the protagonist or an interaction between the two factors, *p*s > 0.15.

**FIGURE 1:**
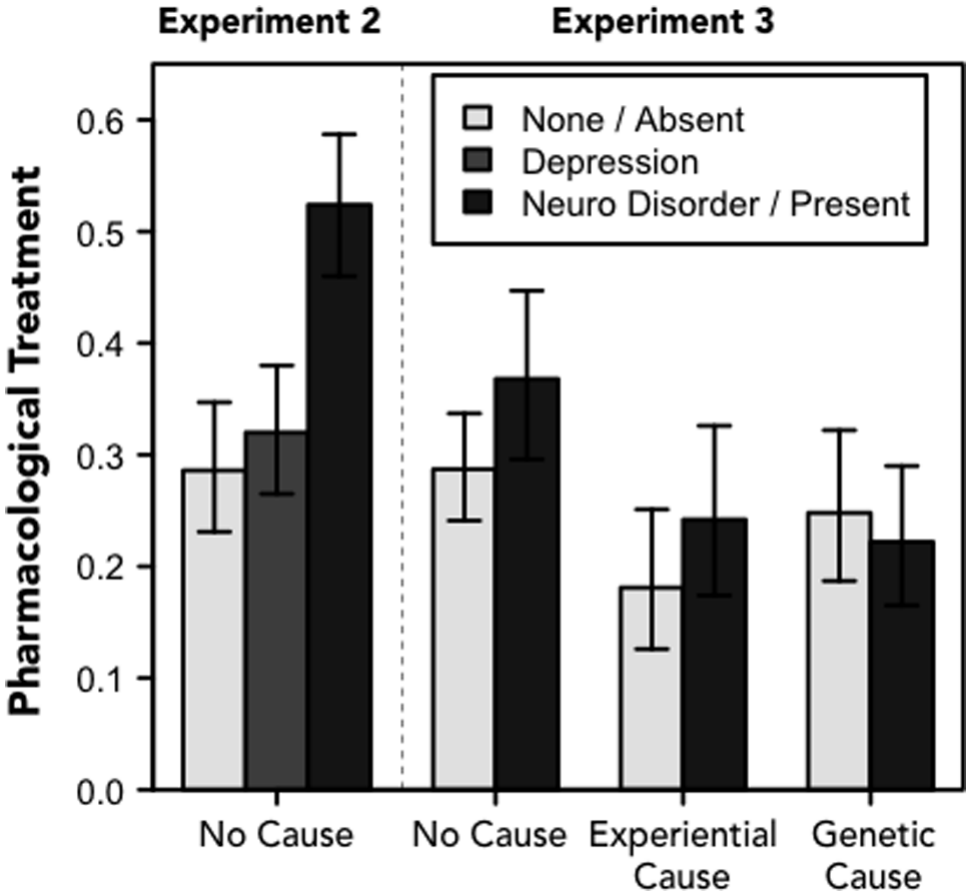
**Treatment.**Proportions of participants who suggested pharmacological treatment for the protagonist in Experiments 2 (left cluster of three bars) and 3 (6 rightmost bars). For Experiment 2, the legend refers to one of there frames; for Experiment 3, the legend refers to whether the narrative included information about the physiological basis of depression. Error bars denote 95% confidence intervals.

As expected, participants were more likely to suggest a pharmacological approach to treatment when they received the “neurological disorder” frame (52%) compared to when the symptoms were identified as resulting from “depression” (32%) or when they were unlabeled (29%), χ^2^(2) = 29.837, *p* < 0.001 (see **Figure 1**). There was no effect of the gender of the protagonist, as a main effect or as an interaction with the labeling manipulation, *p*s > 0.2.

In Experiment 3, we did not expect to find systematic variability in whether participants thought the protagonist should seek treatment because the protagonist was identified as suffering from depression for all participants. However, we did expect to find differences in the kinds of treatment that participants’ thought would be most appropriate as a function of the experimental manipulations. We predicted that participants would be more likely to suggest pharmacological treatment when the narrative included information about the neurological basis of depression and when “genes” were identified as a likely causal contributor to the person’s disorder. We found partial support for these hypotheses.

As expected, Experiment 3 revealed no differences in the proportion of participants who suggested that the protagonist seek help (97% overall) by the experimental manipulations. We did, however, find differences in treatment suggestions by causal explanation, χ^2^(2) = 6.374, *p* = 0.041, and an interaction between causal explanation and neurobiological description, χ^2^(2) = 8.004, *p* = 0.018 (see **Figure 1**). There were no effects of the gender of the protagonist on treatment suggestions, *p*s > 0.8.

As expected, participants were more likely to suggest pharmacological treatment when they were given a neurological description of depression overall. However, counter to what we predicted, the results revealed that including a causal explanation (either type) made people *less* likely to suggest medication – especially when the protagonist was described as having a negative early life experience.

There are at least two possible explanations for this seemingly counter-intuitive finding. One is that identifying the protagonist as suffering from “depression” at the beginning of the narrative overpowered the influence of subsequent information about a genetic basis for the disorder. A second possibility is that people may not associate genetic disorders with medication. People may be able to imagine how psychological treatment could be appropriate for someone dealing with depression that results from a negative life experience, but they do not seem to think of medication as a more suitable treatment for depression that results from a genetic predisposition for the condition.

Framed differently, we did not find a simple relationship between including biological information (describing a neurological basis or identifying a genetic contribution for the disorder) and endorsement of pharmacological treatment. Instead, the results suggest a connection between thinking about the efficacy of pharmacological treatment when the neurological basis of depression was emphasized but not when a genetic basis for the condition was highlighted. However, it should be noted that there are other biologically based treatments (e.g., gene therapy) that may be more congruent with an account of depression that is grounded in genetics, which participants may be more likely to support after reading about the heritability of depression.

### Perceptions of the Protagonist

In Experiment 1, we found no influence of participants’ diagnosis of the protagonist or the gender of the protagonist on the measures of empathy, *F*[1,183] = 0.021, *p* = 0.885, or responsibility, *F*[1,183] = 0.205, *p* = 0.651. This may have been due to the lack of variability in treatment suggestions or it may reflect a genuine lack of a relationship between how the symptoms are labeled and the degree to which people empathize with and attribute responsibility for someone experiencing those symptoms.

**FIGURE 2:**
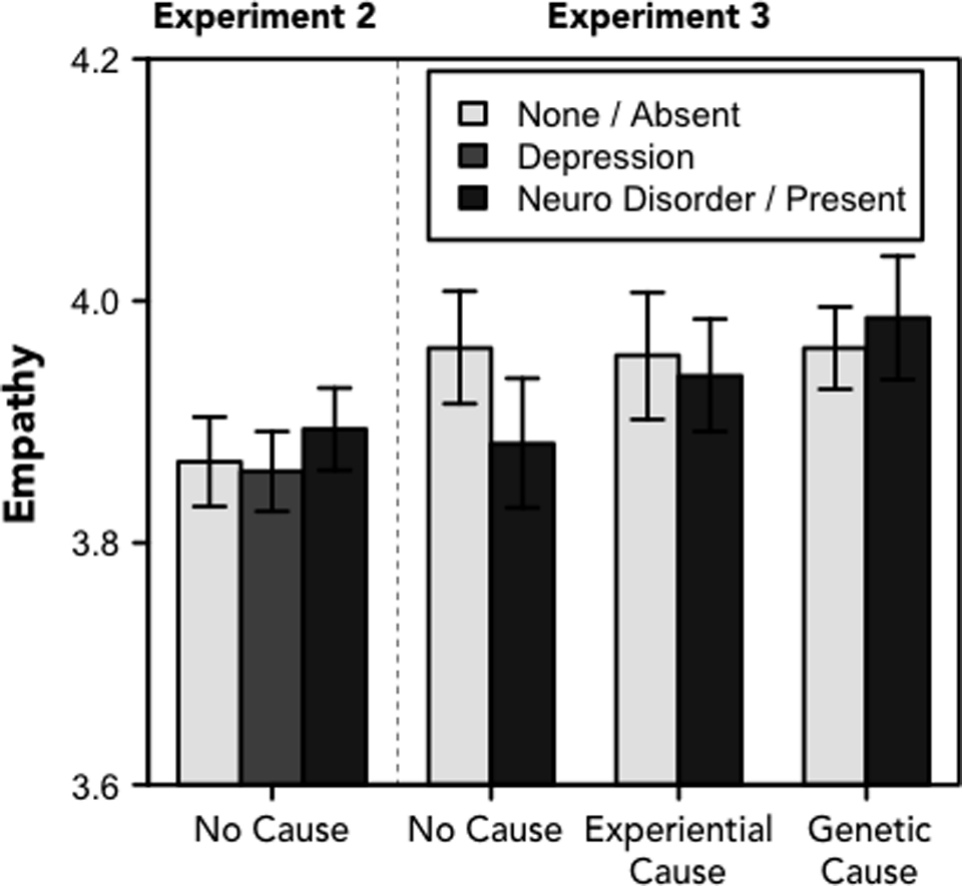
**Empathy.**Ratings of empathy toward the protagonist (range: 1–5). For Experiment 2, the legend refers to one of there frames; for Experiment 3, the legend refers to whether the narrative included information about the physiological basis of depression. Error bars denote standard errors of the means.

Experiments 2 and 3 suggest support for the latter explanation with respect to empathy: in both follow-up experiments, we found no effects of the experimental manipulations on the measure of empathy, *F*s < 1.65, *p*s > 0.2 (see **Figure 2**). That is, different diagnoses, labels, and causal explanations for depressive symptoms did not seem to affect the extent to which people empathized with the person experiencing the symptoms. Although this finding is inconsistent with one of the goals of a messaging campaign that seeks to promote empathy and decrease stigma by highlighting the biological basis of depression (Sartorius, 1997; US Department of Health and Human Services, 2003), these results do not suggest a cost to such labels, as has been found in some previous work (e.g., Phelan et al., 2006; Deacon and Baird, 2009; Dar-Nimrod and Heine, 2011; Lebowitz et al., 2013; Lebowitz and Ahn, 2014).

**FIGURE 3:**
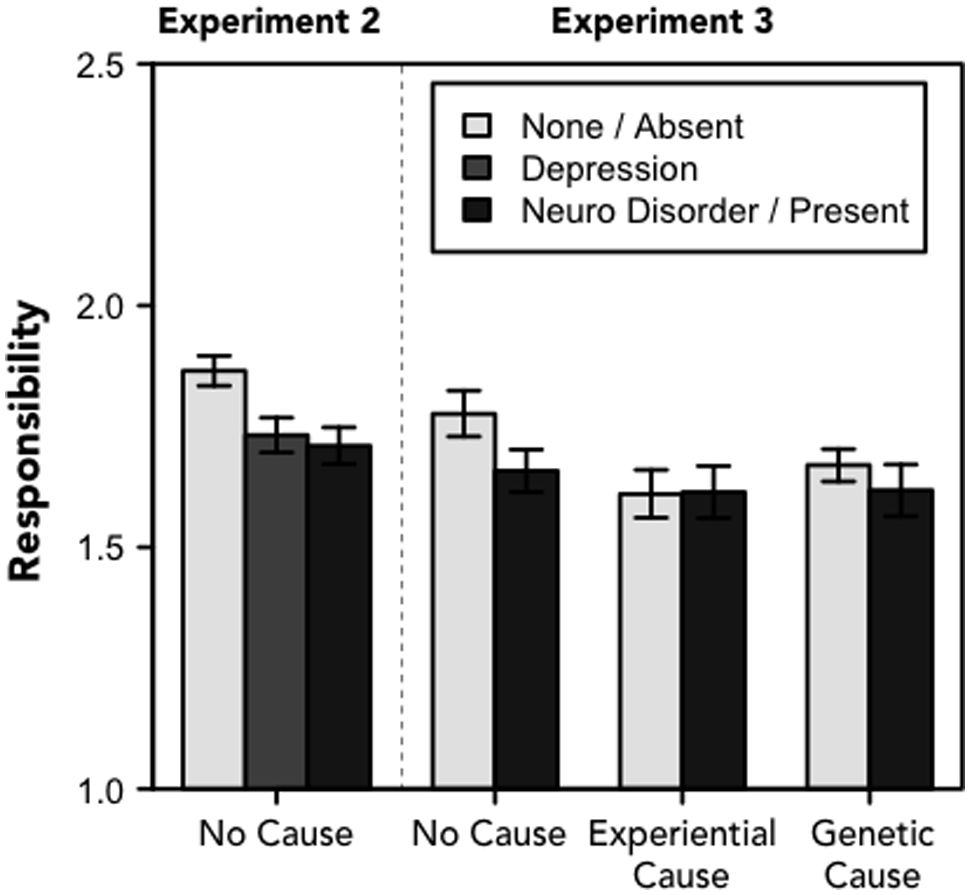
**Responsibility.**Perceptions of the protagonist’s personal responsibility for their symptoms (range: 1–3). For Experiment 2, the legend refers to one of there frames; for Experiment 3, the legend refers to whether the narrative included information about the physiological basis of depression. Error bars denote standard errors of the means.

However, we did find effects of the experimental manipulations on the measure of personal responsibility in Experiments 2 and 3 (see **Figure 3**). In Experiment 2, both the “neurological disorder” (*M* = 1.71), *t*[565] = 3.170, *p* = 0.002, *d* = 0.256, and “depression” labels (*M* = 1.73), *t*[566] = 2.793, *p* = 0.005, *d* = 0.229, led people to attribute less responsibility to the protagonist than the description of the symptoms alone (*M* = 1.86), *F*[2, 841] = 5.750, *p* = 0.003, η*^2^* = 0.013. There was no difference in ratings of responsibility between the two labeled conditions, *t*[557] = 0.430, *p* = 0.667. There was no effect of gender on perceptions of responsibility, *F*s < 1, *p*s > 0.4.

In Experiment 3, there was a marginal main effect of causal explanation, *F*[2,1090] = 2.551, *p* = 0.079, η*^2^* = 0.005. Participants attributed slightly less responsibility to the protagonist when he or she was described as having a negative early life experience (*M* = 1.61) or when depression was described as heritable (*M* = 1.66); participants attributed more responsibility to the protagonist when the narrative lacked a causal explanation (*M* = 1.72). There was no difference in attributions of responsibility as a function of the presence (*M* = 1.63) or absence (*M* = 1.68) of a neurological description, *F*[1,1090] = 2.010, *p* = 0.157; nor were there effects of the gender of the protagonist or two- or three-way interactions between these factors, *F*s < 2, *p*s > 0.15.

These results provide partial support for the view that perceptions of responsibility are malleable and may be affected by a labeling manipulation (or including a causal explanation). However, our results differ from what has been proposed by health practitioners, as there was no difference between the “neurological disorder” and “depression” labels in Experiment 2 and no difference between conditions that included or omitted more elaborate descriptions of the neurobiology of depression in Experiment 3 (Sartorius, 1997; US Department of Health and Human Services, 2003). Instead, these data suggest that providing any label for a diverse set of symptoms might serve to decrease attributions of responsibility. In other words, the act of classification itself, of linking these different symptoms into an overarching explanatory schema, may be sufficient to reduce the sense that the individual is responsible for their behavioral and emotional states.

### Attitudes toward Depression

**FIGURE 4:**
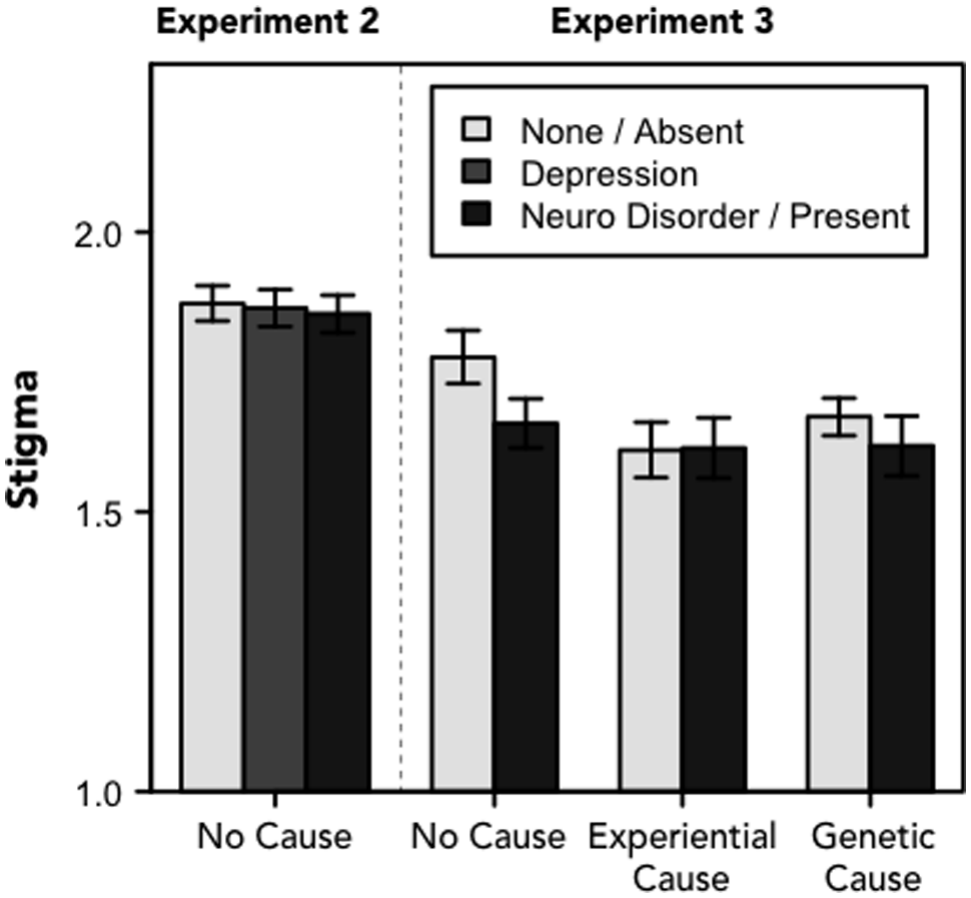
**Stigma.**Stigma toward depression (range: 1–5). For Experiment 2, the legend refers to one of three frames; for Experiment 3, the legend refers to whether the narrative included information about the physiological basis of depression. Error bars denote standard errors of the means.

In Experiment 1, we found no relationship between participants’ patterns of diagnosis and their feelings of stigma toward the disorder or their view of depression as a disease. Results of Experiments 2 and 3 suggest that the degree to which people stigmatize the disorder is relatively stable, as this measure was not influenced by the experimental manipulations in either follow-up study, *F*s < 1.6, *p*s > 0.2 (see **Figure 4**). This is consistent with recent polls and empirical work showing that recognition of the neurobiological basis of depression does not necessarily decrease the stigma associated with the condition (Pescosolido et al., 2010; Lebowitz and Ahn, 2014).

**FIGURE 5:**
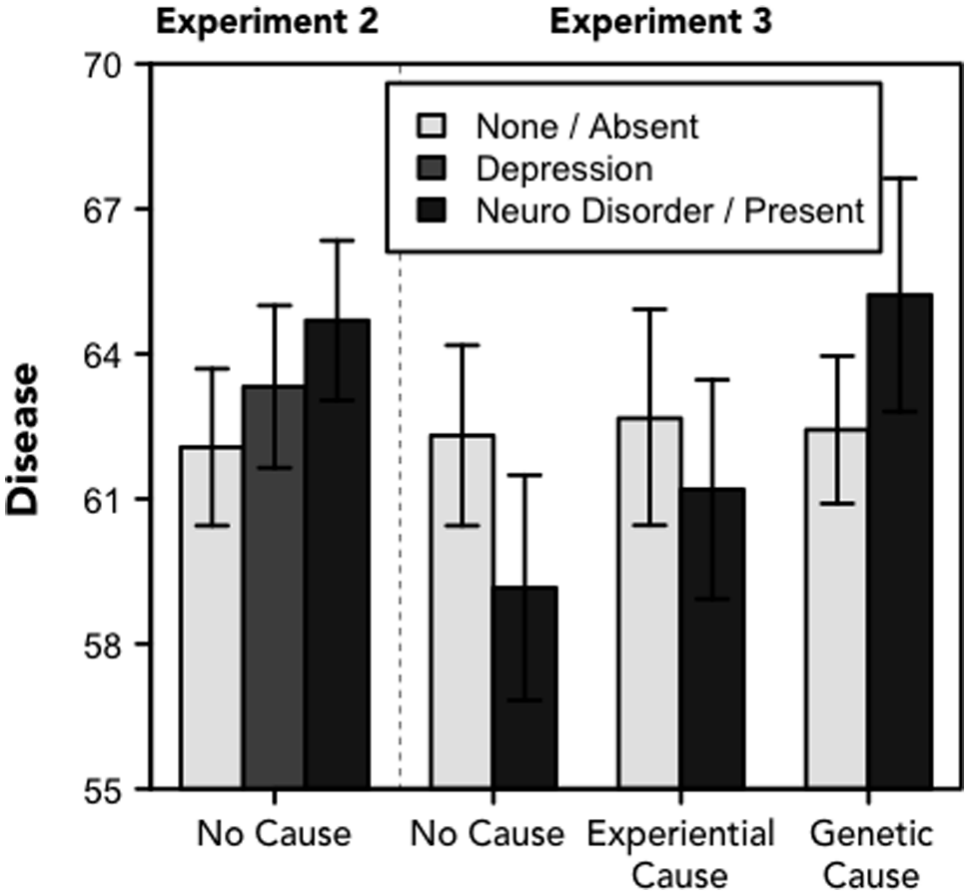
**Disease.**Perceptions of depression as a disease (range: 0–100). For Experiment 2, the legend refers to one of there frames; for Experiment 3, the legend refers to whether the narrative included information about the physiological basis of depression. Error bars denote standard errors of the means.

However, in Experiment 2 conceptions of depression as a disease were influenced by both the labeling manipulation and the gender of the protagonist (see **Figure 5**). An interaction between the label and the gender of the protagonist suggested that the labels affected conceptions of the protagonist when the protagonist was female but not male, *F*[2,841] = 3.509, *p* = 0.030, η*^2^* = 0.008. Participants were least likely to think of depression as a disease when the female protagonist’s symptoms were described without a label (*M* = 57.22); they were more likely to think of depression as a disease when the symptoms followed the “depression” (*M* = 65.38) or “neurological disorder” label (*M* = 68.15), *F*[2,278] = 3.909, *p* = 0.021, η*^2^* = 0.027. The difference between the no label and “neurological disorder” conditions was significant (at the Bonferroni-corrected *α* = 0.017 level), *t*[185] = 2.712, *p* = 0.007, *d* = 0.390. Differences between the “no label” and “depression” conditions and between the “neurological disorder” and “depression” conditions were not statistically significant, *t*s < 2, *p*s > 0.05. On the other hand, participants reported similar conceptions of depression after reading about a male protagonist (*M*s = 62.82, 62.28, and 64.24 in the “neurological disorder,” “depression” and no label conditions, respectively), *F*[2,563] = 0.257, *p* = 0.773.

The gender difference may result from women being stereotypically viewed as less agentic in general than men (Eagly, 1987) and less able to regulate their emotions. Therefore people might be less likely to think of depression as a disease when the symptoms are described in the context of a female protagonist.

In Experiment 3, we found no effects of the experimental manipulations on conceptions of depression as a disease, *F*s < 1.6, *p*s > 0.2 (see **Figure 5**). Despite recent survey data indicating that views of depression as a disease are on the rise (consistent with the efforts of messaging campaigns; Pescosolido et al., 2010), we did not find that describing a protagonist as suffering from a neurological disorder (or including a more elaborate description of the physiological basis of depression) made people more likely to view depression as a disease. Instead, we found some indication, in the context of a female protagonist, that people were more likely to view someone who is labeled with any clinical condition (“depression” or “neurological disorder”) as suffering from a disease relative to someone whose symptoms are not classified.

### Individual Differences

As predicted, in all three experiments we found that participants’ personal history with mental illness, their gender, educational history, and their political ideology were systematically related to perceptions of the protagonist and conceptions of depression. We did not find relationships between the participants’ age and the dependent measures. **Table 3** displays relationships between the individual differences measures and the dependent measures from data aggregated across all three experiments.

**Table 3:** Individual differences.

	Treatment	Empathy	Responsibility	Disease	Stigma
History of depression	38% Rx [0.35, 0.41]	4.17 (0.52)	1.64 (0.62)	67.55 (27.60)	1.73 (0.49)
No history of depression	24% Rx [0.21, 0.27]	3.63 (0.55)	1.79 (0.59)	57.42 (26.57)	2.08 (0.53)
Female	31% Rx [0.28, 0.34]	4.01 (0.56)	1.65 (0.63)	66.84 (28.15)	1.78 (0.50)
Male	32% Rx [0.29, 0.35]	3.81 (0.62)	1.76 (0.58)	58.58 (26.34)	2.02 (0.55)
Pol affiliation					
Democrat	33% Rx [0.29, 0.36]	3.94 (0.60)	1.68 (0.61)	64.62 (27.15)	1.88 (0.54)
Independent	28% Rx [0.24, 0.31]	3.89 (0.62)	1.72 (0.62)	60.15 (27.55)	1.93 (0.52)
Republican	35% Rx [0.31, 0.39]	3.88 (0.58)	1.74 (0.60)	62.58 (28.07)	1.89 (0.55)


*Judgments regarding the protagonist and depression as a function of participants’ history with depression, gender, and political affiliation with data aggregated from Experiments 1, 2, and 3. Percentages and confidence intervals are shown for treatment suggestions; means and standard deviations are shown for the other four measures.*

Since the individual difference measures were highly correlated with one another in some cases (e.g., female participants were more likely to report a history of depression, χ*^2^*(1) = 4.614, *p* = 0.032) we fit multiple regression models to suggested treatments (logistic), empathy, attributions of responsibility, judgments of depression as a disease, and stigmatized attitudes toward depression. Participants who reported that their sex was neither male nor female or who declined to respond about their history with depression were excluded from these analyses.

Participants with a history of depression responded differently than participants who did not report a history of depression on nearly every dependent measure. They were more likely to suggest that the protagonist treat their symptoms with medication, β = 0.657, *SE* = 0.108, *p* < 0.001, more likely to empathize with the protagonist, β = 0.832, *SE* = 0.040, *p* < 0.001, and less likely to attribute responsibility to the protagonist for their experience, β = -0.185, *SE* = 0.044, *p* < 0.001. They were also more likely to think of depression as a disease, β = 0.267, *SE* = 0.043, *p* < 0.001, and less likely to hold stigmatizing attitudes toward the condition, β = -0.530, *SE* = 0.041, *p* < 0.001. These findings are generally consistent with prior work showing how first-hand experience with depression affects the way people think about the condition (e.g., Lebowitz et al., 2013).

We also found differences by the gender and ideology of participants. Women and liberals reported more empathy toward the protagonist, β = 0.125, *SE* = 0.039, *p* < 0.001 and β = 0.128, *SE* = 0.020, *p* < 0.001, attributed less responsibility to him or her, β = -0.131, *SE* = 0.044, *p* = 0.003 and β = -0.118, *SE* = 0.022, *p* < 0.001, were less likely to hold a stigmatizing view of the condition, β = -0.301, *SE* = 0.041, *p* < 0.001 and β = -0.173, *SE* = 0.020, *p* < 0.001, and were more likely to think of depression as a disease, β = 0.216, *SE* = 0.043, *p* < 0.001 and β = 0.182, *SE* = 0.021, *p* < 0.001. Neither the gender of the participant nor their political ideology affected their treatment suggestion.

These finding are consistent with recent research on the relationship between gender, political ideology and folk psychiatric reasoning in other domains like obesity and addiction (e.g., Flusberg et al., 2015; Thibodeau et al., 2015). In addition, the influence of political ideology is consistent with a conservative worldview, where people are viewed as especially accountable for their behavior and feelings (e.g., Skitka et al., 2002).

In addition, we found that participants with more education attributed less responsibility to the protagonist, β = -0.047, *SE* = 0.022, *p* = 0.028, and were less likely to hold a stigmatized view of depression, β = -0.045, *SE* = 0.020, *p* = 0.024, also consistent with prior work (e.g., Corrigan and Watson, 2007). There were no relationships between participants’ education level and treatment suggestions, empathy, or conceptions of depression as a disease.

Finally, we considered the possibility of a gender congruence effect (e.g., Kulik and Holbrook, 2000): would females respond more favorably to a female protagonist; would males respond more favorably to a male protagonist? We found that the relationship between the gender of the protagonist and participant impacted judgments of depression as a disease, *F*[1,2177] = 4.22, *p* = 0.032, but not treatment suggestions, empathy, attributions of responsibility, or stigma, *F*s < 2.6, *p*s > 0.1. As noted above, females were more likely than males to consider depression a disease. This was especially true when the protagonist was female as compared to when the protagonist was male, *t*[1048] = 2.154, *p* = 0.031 (see **Table 4**) and reveals partial support of a gender congruence effect – for women but not men.

**Table 4:** Gender congruence.

Participant’s gender	Protagonist’s gender	Treatment	Empathy	Responsibility	Disease	Stigma
Female	Female	30% [0.26, 0.35]	4.01 (0.54)	1.63 (0.66)	68.92 (26.82)	1.78 (0.50)
Female	Male	32% [0.28, 0.36]	4.00 (0.58)	1.66 (0.61)	65.16 (29.08)	1.78 (0.50)
Male	Female	34% [0.29, 0.39]	3.87 (0.64)	1.69 (0.57)	58.23 (27.17)	1.97 (0.55)
Male	Male	30% [0.30, 0.34)	3.77 (0.60)	1.81 (0.57)	58.84 (25.74)	2.05 (0.54)


*Judgments regarding the protagonist and depression as a function of the relationship between the gender of the participant and protagonist with data aggregated from Experiments 1, 2, and 3. Percentages and confidence intervals are shown for treatment suggestions; means and standard deviations are shown for the other four measures.*

## Discussion

In three experiments, we tested how labels and explanations for a person’s depressive symptoms affected views of this person and conceptions of depression. Despite the laudatory goals of recent messaging campaigns, we find little evidence that emphasizing the neurobiological basis of depression promotes empathy, encourages people to view depression as a disease, or that such a change leads to a reduction in attributions of responsibility or stigmatizing attitudes. Emphasizing the neurobiological underpinning of depression had the biggest effect on participants’ treatment suggestions – leading them to suggest medication and therapy in most cases, rather than therapy alone. This represents a valuable finding, as many studies have found that the combination of psychotherapeutic and biological treatments represents the most effective means of dealing with a variety of mental disorders, including depression (e.g., Elkin et al., 1989; Bowers, 1990). That is, one positive effect of describing depression in biological terms is that it seems to shift peoples’ conception of how to treat depression to be more consistent with treatment recommendations that have emerged from the medical literature.

The results of all three experiments suggest that the degree to which people view depression as a disease seems to anchor a spectrum of depression-related attitudes. This is consistent with other research on folk psychiatric reasoning, which suggests that the biological or medical dimension plays a key role in how people conceptualize and make decisions with respect to complex health conditions (Haslam, 2005; Lebowitz et al., 2013, 2014; Lebowitz and Ahn, 2014; Flusberg et al., 2015). In particular, when the depression schema conforms to a disease model, people tend to feel more empathy toward those suffering from depressive symptoms, hold fewer stigmatizing attitudes toward the condition, and attribute less responsibility to the individual for the current situation they find themselves in.

While the labeling and causal explanation manipulations did little to shift people’s beliefs about whether or not depression should be thought of as a disease, we found strong and consistent effects of a variety of individual difference measures on this dimension of the depression schema. Specifically, people with a history of depression, females, and liberals were more likely to view depression as a disease. Such a view was related, in turn, with more empathy, less attribution of responsibility, and less stigma. This is largely consistent with research on the factors that influence conceptions of other conditions like addiction (Flusberg et al., 2015) and obesity (Thibodeau et al., 2015*).* This set of findings is important because it reveals both the wide range of variability in the depression schemas people have in mind, as well as the factors that may shape the schema any individual holds in particular. Identifying the influence of the individual difference measures may also be valuable to clinicians, educators, and policy makers when designing educational interventions related to the nature and treatment of depression.

Interestingly, prior work has in fact found that framing manipulations can impact the extent to which people hold a more biological or “medicalized” view of certain mental disorders (Lebowitz and Ahn, 2014; Lebowitz et al., 2014). It is possible that certain design features of our experiments limited the efficacy of the labels and explanations used in the present set of studies. For instance, in all three experiments the symptoms were described in detail at a psychological or behavioral level (e.g., trouble sleeping), which may overpower a subtle framing manipulation or a discussion of the neurological basis of the disorder. Similarly, in Experiment 3 the protagonist was identified as suffering from depression even in conditions in which the neurobiological basis of depression was detailed, which may have constrained the influence of the explanation.

As a result, we think it would be premature to conclude that simply emphasizing the neurobiological underpinning of depression is a poor strategy for changing certain attitudes toward depression. Indeed, evidence from messaging campaigns in the context of other health issues like anorexia suggest that emphasizing the neurobiological component of the disorder can be an important tool for promoting the view of psychological disorders as a disease (e.g., Crisafulli et al., 2008. See also Lebowitz et al., 2014). However, further work is needed to determine exactly how to use this information effectively in the context of depression.

For instance, it may be important to describe the neurobiology of depression in relation to the protagonist in particular. In the present studies, the neurobiology of depression was discussed at an abstract level: “Researchers have found a number of abnormalities in the neurobiology of people with depression.” However, it may be more valuable to make such statements at a personal level: “Doctors have found a number of abnormalities in the function of Mark/Jenny’s brain that affect his/her ability to regulate his/her mood.” Alternatively, or in addition, it may be important to explicitly identify depression as a disease (e.g., “depression is a brain disease”) rather than simply frame a set of symptoms as the result of a relatively ambiguous “neurological disorder.” These open questions represent important opportunities for future work (but see Lebowitz and Ahn, 2014; Lebowitz et al., 2014; Flusberg et al., 2015).

Importantly, we do not find evidence of a cost for emphasizing the neurobiological underpinning of depression that has been found or theorized by others (Phelan et al., 2006; Deacon and Baird, 2009; Dar-Nimrod and Heine, 2011; Lebowitz et al., 2013; Lebowitz and Ahn, 2014). Reading that the protagonist was suffering from a neurological disorder or an extended discussion of the neurobiology of depression did not lead to an increase in stigma or blame.

One additional take-away from these studies is that people may view aspects of depression differently based on the gender of the person showing symptoms of the disorder. For instance, we found that the framing manipulation was more effective in the context of a female protagonist and that people were less likely to attribute responsibility to a female protagonist suffering from a disorder (either “depression” or a “neurological disorder”). This may be because males are viewed as more agentive in general (e.g., Eagly, 1987) or because depression is more common among women (Piccinelli and Wilkinson, 2000). As a result, people may be more compassionate in their evaluation of a description of a female suffering from depression. However, on most measures, we found no differences in how people evaluated a male and female protagonist who was described as suffering from depression.

In sum, this work represents a valuable step toward understanding lay conceptions of depression, reveals an initial empirically grounded effort to characterize a lay schema for depression, and offers empirical evidence for some of the key important factors that may influence these conceptions. These findings are important not only in the context of clinical health communications, but also for cognitive scientists interested in how schematic knowledge guides reasoning and decision-making in complex domains like mental illness.

## Conflict of Interest Statement

The authors declare that the research was conducted in the absence of any commercial or financial relationships that could be construed as a potential conflict of interest.
