# Supplementary material for: The Depression Schema: How Labels, Features, and Causal Explanations Affect Lay Conceptions of Depression
Source: Front Psychol. 2015 Nov 17;6:1728. doi: 10.3389/fpsyg.2015.01728 (PMC4648076; doi:10.3389/fpsyg.2015.01728)
Supplement: Supplementary file 1 [file Table_1.DOCX]

Supplementary Material

The depression schema:

How labels, features, and causal explanations affect lay conceptions of depression

Paul H. Thibodeau^1^*, Mira J. Fein^1^, Elizabeth S. Goodbody^1^, Stephen J. Flusberg^2^

^1^Department of Psychology, Oberlin College, Oberlin, OH, USA

^2^Department of Psychology, Purchase College, Purchase, NY, USA

*** Correspondence:** Paul Thibodeau: paul.thibodeau@oberlin.edu

# Compressive Model

To unify these findings, confirm the effects of the experimental manipulations, and test for effects of the individual difference measures, we fit a structural equation model using standard Maximum Likelihood Estimation to the data from Experiment 2. We omitted data from participants who identified their gender as “other” (*n* = 7) and from participants who chose not to answer whether they had a history of depression (*n* = 18), leaving data from 822 participants for the SEM (97% of the full data set).

The first model included individual difference measures (history with depression, gender, and political ideology), participants’ attitudes toward the protagonist (i.e. empathy, attributions of responsibility, whether treatment should include medication), and participants’ attitudes toward depression generally (i.e. depression as a disease, stigma). The best fitting model for these data is shown in Figure 6, *χ^2^*[df=39, N=822] = 346.913, *p* < .001 (*CFI*=.682; *RMSEA*=.098, *SRMR*=.079, *AIC*=400.913, *BIC*=85.155).

Supp Fig. 1

The path analysis shows predictable covariation between viewing depression as a disease, attributions of responsibility, feelings of empathy, and stigmatizing attitudes. Namely, the more people view depression as a disease, the more empathy they feel towards (and the less responsibility they attribute to) those experiencing symptoms of depression. People also hold lower stigmatized attitudes towards depression to the extent they think about it as a disease. This model also confirms previously discussed findings about the relationship between the individual difference and attitudinal measures; conservatives, males, and those without any personal history of depression are less likely to think of depression as a disease and thus more likely hold stigmatized attitudes towards the condition and less likely to feel empathy towards those experiencing symptoms of the disorder.

To test for effects of the framing and gender manipulations, we analyzed whether the labeling manipulation affected each of the dependent measures. We found that the labels significantly modulated perceptions of the protagonist’s responsibility for their symptoms and the recommended course of treatment, consistent with the analyses reported previously. The labeling manipulation did not directly affect conceptions of depression as a disease, stigma toward the condition, or empathy toward the protagonist. In addition, we found that adding a predictor for the gender of the protagonist did not significantly improve the fit of the model. Updating the model to include the effect of the labels on perceptions of responsibility and treatment suggestions represented significant improvement, *χ^2^*[df=36, N=822] = 309.782, *p* < .001 (*CFI*=.717; *RMSEA*=.096, *SRMR*=.076, *AIC*=369.782, *BIC*=68.159).

In sum, the SEM provides important theoretical and practical insights about how various depression-related attitudes relate to one another. It represents one way of conceptualizing and visualizing relationships between elements of a folk schema for depression. Of particular value, it suggests that the degree to which people’s depression schema is organized around a disease model plays a central role in how people think about the disorder. This in turn is influenced by a person’s history with depression, their gender, and their political ideology, but not by the label or diagnosis they are provided with or the gender of the person described in the narrative.

**Supplementary Figure 1.** **Path Analysis.** A structural equation model of the relationship between the labeling manipulation, individual difference measures, attitudes toward depression, and attitudes toward the protagonist. Asterisks denote statistically significant coefficients, **p* < .05, ***p* < .01, ****p* < .001.
